# A Competency Framework for Developing Global Laboratory Leaders

**DOI:** 10.3389/fpubh.2019.00199

**Published:** 2019-08-20

**Authors:** Adilya Albetkova, Evelyne Chaignat, Philippe Gasquet, Martin Heilmann, Jocelyn Isadore, Aftab Jasir, Barbara Martin, Burton Wilcke

**Affiliations:** ^1^Centers for Disease Control and Prevention, Atlanta, GA, United States; ^2^World Health Organization, Lyon, France; ^3^Association of Public Health Laboratories, Silver Spring, MD, United States; ^4^European Centre for Disease Prevention and Control, Stockholm, Sweden; ^5^World Organisation for Animal Health, Paris, France

**Keywords:** laboratory system, leadership, health system, one health, competency, management, program, leaders

## Abstract

Building sustainable national health laboratory systems requires laboratory leaders who can address complex and changing demands for services and build strong collaborative networks. Global consensus on laboratory leadership competencies is critically important to ensure the harmonization of learning approaches for curriculum development across relevant health sectors. The World Health Organization (WHO), the Food and Agriculture Organization of the United Nations (FAO), the World Organisation for Animal Health (OIE), the European Centre for Disease Prevention and Control (ECDC), the U.S. Centers for Disease Control and Prevention (CDC), and the Association of Public Health Laboratories (APHL) have partnered to develop a Laboratory Leadership Competency Framework (CF) that provides a foundation for the Global Laboratory Leadership Programme (GLLP). The CF represents the first global consensus from multiple disciplines on laboratory leadership competencies and provides structure for the development of laboratory leaders with the knowledge, skills and abilities to build bridges, enhance communication, foster collaboration and develop an understanding of existing synergies between the human, animal, environmental, and other relevant health sectors.

## Introduction

Laboratories are an essential and fundamental part of health systems and play a vital role in the detection, diagnosis, and control of diseases (1). However, reliable laboratory services continue to be limited in many low- and middle-income countries (2, 3). Although there have been positive examples of laboratory responses to outbreaks (4–6), a number of well-documented events including some at the convergence of human, animal and environmental health have shown how a lack of robust laboratory systems can impede disease detection, control and prevention efforts (7–9). These circumstances highlight the importance of building sustainable national health laboratory systems including strong linkages and cooperation between sectors and within the health system. Such systems require laboratory leaders who can lead and manage laboratories under any circumstances including potentially turbulent environments, to advocate for appropriate laboratory diagnostics, and to build strong collaborative networks with relevant sectors at every level of the health system in support of better health for people, animals, and their shared environment (10).

Although much progress has been made in the development of leadership competencies in the field of health worldwide (11, 12), limited attention has been paid to laboratory leaders specifically. This lack of specialized training for laboratory professionals in the areas of leadership and management has been well-articulated (13, 14). Several initiatives have been launched, but these only partially address the identified gaps (13, 15–18). These initiatives also identify the need for a comprehensive, competency-based framework that provides the foundation for training and education of laboratory leaders globally. Therefore, building a global consensus on laboratory leadership competencies is critically important to ensure the harmonization of learning approaches for curriculum development across human, animal, environmental and other relevant health sectors.

Recognizing this need, the World Health Organization (WHO), the Food and Agriculture Organization of the United Nations (FAO), the World Organisation for Animal Health (OIE), the European Centre for Disease Prevention and Control (ECDC), the Centers for Disease Control and Prevention (CDC), and the Association of Public Health Laboratories (APHL) have partnered to develop a Laboratory Leadership Competency Framework (CF) that provides a foundation for the upcoming Global Laboratory Leadership Programme (GLLP) (19).

This CF will facilitate the development of training programs for laboratory leaders to acquire the knowledge, skills and abilities to successfully build sustainable national health laboratory systems and foster collaboration across all components of the health system. Acknowledging the interrelatedness of human, animal and environmental health, this CF specifically embodies a One Health approach and considers a “National Health Laboratory System” as a network including human, animal, environmental, agricultural, food, aquatic and chemical laboratories in support of health systems.

Our paper provides an overview of CF designed to outline essential competencies needed for laboratory leaders to build sustainable national health laboratory systems. In the context of CF, the “laboratory leader” is defined as an individual laboratory subject matter expert responsible for managing finance, motivating staff, advocating for the laboratory, building relationships with external partners and navigating legislative processes.

### Development Process

The CF was developed through a consensus process involving subject matter experts from the aforementioned GLLP partners. In October 2017, the partners established the GLLP Competency Framework Development Workgroup comprised of laboratorians as well as learning experts from each organization. The following nine competencies were selected through extensive discussion and debate considering the relevant literature [(20, 21) and World Health Organization, unpublished report of the WHO laboratory leadership and management training programme meeting. Lyon, France 12-13 May 2011], each partner's expertise, experience and lessons learned in the field, as well as a consideration of the past, present and future role of health laboratories across the globe:

1. Laboratory system
2. Leadership
3. Management
4. Communication
5. Quality management system
6. Biosafety and biosecurity
7. Disease surveillance and outbreak investigation
8. Emergency preparedness, response and recovery
9. Research

From November 2017 to March 2018, the Workgroup designed the structure of the framework, developed descriptions of each competency and defined competency domains, subdomains, and areas of activity. The action verbs that describe the levels of proficiency for each area of activity were developed based on the Structure of the Observed Learning Outcome (SOLO) (22) and Bloom's (23) taxonomies. Some modifications of the verbs were incorporated to represent learning outcomes specifically related to leadership. Performance activities are associated with levels of proficiency and are presented at three levels—developing, skilled, and expert.

Multisectoral laboratory workforce development is essential and the use of common competencies across disciplines and organizations can facilitate communication and cooperation and allow for career development and growth. The CF specifically integrates competencies applicable to laboratory experts and current leaders and managers from different sectors addressing and aligning disparate terminology. This CF can therefore be used by national authorities from all sectors and disciplines: policy makers, regulatory agencies, educational institutions, and other stakeholders including donors, non-governmental organizations (NGOs), and the private sector (24).

### Competencies Description

The CF was designed to outline the nine essential competencies needed for laboratory leaders to build sustainable national health laboratory systems that improve disease and chemical/residue detection, control and prevention efforts. Table 1 provides an overview of the CF design. Competency areas may be applied in all sectors of the national health laboratory system, at the system level and/or the facility level and duplication of areas across competencies is intentional to facilitate flexible use of the framework. Descriptions of each competency follow:

**Table 1:** Laboratory leadership competency framework design.

Each competency is structured as follows: **Competency:** A combination of the knowledge, skills, and abilities that are critical to perform a task effectively (for example, “3.Management”).		
**Competency domain:** A discrete component of a competency (for example, “3.2 Resource management”).		
**Subdomain:** A subcomponent of a domain (for example, “3.2a. Budgeting and financial management”).		
**Area:** Competency domains and subdomains are broken down further Into areas of activity (for example, “3.2.1. Laboratory budget”,”3.2.2. Financial auditing process”, “3.2.3. Financial resource utilization”)		
Performance activities are designed in levels according to proficiency, as described below:		
**Levels of Proficiency**		
**Developing:** The individual has advanced knowledge of the principles, concepts and/or methodologies related to the competency as attained through educationor training (e.g., coursework, on-the-job orientation.mentorship, etc.). and is able to perform a range of assignments under supervision, or during mentorship and/or coaching. **Skilled:** The individual analyses and independently applies principles, concepts and/or methodologies related to the competency as attained through education or training and successful experience in a variety of complex assignments.	**Expert:** The Individual has mastered the principles, concepts and/or methodologies related to the competency and has demonstrated significant success in performing the most demanding assignments requiring the competency. Applies innovations in the competency to problem solving and task completion and is able to synthesize, critique or teach the competency and is able to provide coaching and mentoring. For each performance activity, action verbs are standardized according to level of proficiency, as shown below.	
**Action verbs ^a^ by level of proficiency**		
**Developing**	**Skilled**	**Expert**
Define: To determine or identify the essential qualities or meaning of Describe: To represent or give an account of in words (or represent by figure, model, or picture) Identify: To establish the identity of Outline: To indicate the principal features or different parts of List: To make a simple series of words or numerals	Explain: (1) to give the reason for or cause of;(2) to show the logical development or relationships of Analyze: to study or determine the nature and relationship of the parts Apply: to put to use, especially for practical purposes Demonstrate:(1) to prove or make clear by reasoning or evidence;(2) to illustrate and explain, especially with many examples Implement: to give practical effect to and ensure of actual fulfillment by concrete measures	Create: (1) to produce or bring about by a course of action:(2) to produce through skill;(3) to make or bring into existence something new Design: (1) conceive and plan out in the mind;(2) to draw plans for Develop: to set forth or make clear by degrees or in detail Evaluate: to determine the significance, worth or condition of, usually by careful appraisal and study Perform: ^b^ to carry out an action Prioritize: to list or rate in order of priority

a * Definitions for action verbs adapted from Merriam-Webster dictionary (https://www~merriam-webster.com)*.

b * May be used at more than one level of proficiency, depending on context*.

### Competency 1: Laboratory System

A laboratory system and the network of laboratories that make up the system (within a respective jurisdiction, e.g., at the country level) are key to the provision of laboratory services in support of a health system. This competency encompasses the knowledge, skills, and abilities needed to develop, maintain and strengthen a complete and functional health laboratory system capable of producing high quality results with efficient and effective procedures, administration and policies throughout all levels of the health system.

### Competency 2: Leadership

Leadership is essential for success in the fast-paced, changing environment of health laboratory systems. This competency comprises the knowledge, skills and abilities essential for a leader to motivate and inspire a group of people to act toward achieving a common goal. This includes strategic approaches with vision and plans for sustainable and improved success of the laboratories or laboratory systems through the process of positively influencing the actions to attain desired outcomes.

### Competency 3: Management

Strategic management of materials and personnel is necessary for success particularly in an environment where resources may be limited. Without sound management, leadership vision cannot be attained. This competency includes the knowledge, skills, and abilities needed to effectively and efficiently achieve quality laboratory results using available resources. Management of the laboratory may include operational management as well as long-term strategic planning, to achieve the leadership vision.

### Competency 4: Communication

Appropriate communication in all forms is vital for successful leadership and management. This competency encompasses the knowledge, skills, and abilities necessary to communicate laboratory findings and laboratory system related information across sectors and disciplines in a clear and concise manner adjusted to the level and type of audience.

### Competency 5: Quality Management System

The cornerstone of a successful laboratory system is quality results that are reliable, repeatable and timely, and thus allow effective decision-making related to patient care, public health or health policy. The knowledge, skills and abilities to implement and sustain a national quality management system and a culture of quality in laboratory operations are included in this competency.

### Competency 6: Biosafety and Biosecurity

Strong leadership is needed to ensure the necessary safeguards are in place to protect laboratory personnel and the population from biosafety or biosecurity breaches. This competency contains the knowledge, skills and abilities to ensure the laboratory system is operating in a way that optimally minimizes and manages the risks related to biohazards.

### Competency 7: Disease Surveillance and Outbreak Investigation

Laboratory data is essential for accurate disease surveillance, detection and investigation. This competency comprises the knowledge, skills, and abilities required for the on-going, routine management of a health surveillance system including outbreak detection and response, both at the laboratory system level as well as in an individual laboratory.

### Competency 8: Emergency Preparedness, Response and Recovery

Emergencies, both man-made and natural, provide unique challenges to a laboratory system and require sufficient planning and preparation across involved sectors for an adequate and coordinated response. The knowledge, skills and abilities needed to prepare for, respond to and recover from an emergency or other natural or human-caused adverse health event are contained in this competency.

### Competency 9: Research

Although often neglected, laboratory research is a critical part of effective and innovative laboratory management and contributes to the strength of laboratory development and sustainability. This competency comprises the knowledge, skills and abilities needed to plan, conduct and analyze hypothesis-driven investigations that include innovative approaches and methods, testing and evaluation designed to advance health by correlating basic science with clinical, epidemiological and laboratory practice across sectors and disciplines.

A sample of the CF is shown in Table 2. The complete CF can be found here: https://apps.who.int/iris/bitstream/handle/10665/311445/9789241515108-eng.pdf

**Table 2:** Laboratory leadership competency framework sample.

The full Framework can be accessed on the World Health Organization website	
Competency 1. Laboratory System	
Knowledge, skills, and abilities needed to develop, maintain and strengthen a complete and functional national health laboratory system*capable of *producing high quality results using efficient and effective procedures, administration and policies throughout all levels of the health system*.	
^*^A national health laboratory system Is defined as network(s) that Includes human, animal, environmental, agricultural, food, aquatic, and chemical laboratories in support of health systems.	
Domain 1.1. Policy and legal framework	
1.1.1 Organizational structure (see also 1.4.1, 2.1.2)	
	Performance activities
Developing	Outline the organization of the national/regional/multinational/international networks of laboratories
Skilled	Explain the organization of the national/regional/multinational/international networks of laboratories
Expert	Evaluate the organization of the national/regional/multinational/international networks of laboratories
1.1.2 Human-animal-environmental interface	
	Performance activities
Developing	Identify the sectors and disciplines working within the human-animal-environmental interface
Skilled	Explain the various roles of the sectors and disciplines working within the human-animal-environmental interface
Expert	Evaluate collaboration among the various sectors end disciplines working within the human-animal-environmental interface
1.1.3 National policies (see also 5.1.5, 5.3.1, 9.1.3)	
	Performance activities
Developing	List existing national policies that impact laboratory practice
Skilled	Explain the cause and effect of the existing national policies that impact laboratory practice
Expert	Design national policies that maximally support laboratory practice through a consultative process
1.1.4 Legal framework	
	Performance activities
Developing	Identify the legal framework that governs laboratory systems operations
Skilled	Explain the legal framework that governs laboratory systems operations
Expert	Evaluate the legal framework that governs laboratory systems operations

## Discussion

The development of the laboratory leadership CF represents the first global effort involving input and support from multiple organizations and institutions to create a core set of competencies for laboratory leaders working in support of health systems. The CF is designed to allow for the incremental development of skills from the developing to expert levels, has the flexibility to adapt to unique country situations and can be used for leadership development at multiple organizational levels.

The CF can be used at institutional and individual levels for:

- Workforce development: as a standardized reference for laboratory workforce development applicable across national/regional health laboratory systems.
- Program and curricula development: as a foundation for laboratory leadership curricula and programs.
- Specific job descriptions: as guidance for writing standardized job descriptions.
- Needs assessment: as a guidance to develop a tool for self-assessment, observer assessment or a combination of both to identify individual or group needs and guide staff development planning.
- Self-assessment: as guidance for individuals to assess their current level of knowledge, skills and abilities, identify areas in need of improvement and plan for achieving higher levels of proficiency.

The use of the CF across multiple sectors can furthermore encourage development of collaboration and coordination among and across laboratory network and disciplines. The competencies include the skills necessary for laboratory leaders and managers to not only manage the day-to-day workings of a laboratory and laboratory system, but also the multidisciplinary skills needed to move the laboratory or laboratory system to the next level.

Each performance activity within the competencies is specified at three levels of proficiency: developing, skilled and expert. The developing level may be utilized when fostering promising future leaders, whereas the skilled and expert levels may be applied by more experienced professionals. The three-step approach to reaching expert proficiency allows for development of programs appropriate to a wide range of countries with step-wise progress as a goal. The competencies also provide a global perspective recognizing that situations and needs vary considerably between countries. The competencies may be applied to variously structured laboratory systems and may be utilized at the system, organizational or facility level for maximum impact. With the rapid advancement of technology, the need for leaders who can navigate in a changing environment and stay informed and up-to-date is paramount. The CF addresses this issue by emphasizing the importance of research and recognizing that technical competencies are subject to change and require regular updates.

The CF was developed in broad terms to encompass relevant sectors at every level of the health system. The CF addresses environmental aspects but recognizes that the integration of environmental health input has been limited to date. Recognizing that there is not one universal One Health approach but rather an ongoing evolution of One Health approaches, especially when addressing laboratory specific needs, the incorporation of environmental considerations remains ongoing.

### GLLP Development

The CF serves as a foundation for the GLLP, a comprehensive program encompassing the nine laboratory leadership competencies. The six GLLP partners are currently developing the GLLP Learning Package based on the CF. The GLLP will allow countries to adapt programs to meet their exact needs and the GLLP Learning Package will include core course materials and guidance for program development, planning, implementation and evaluation in any country or educational institution in the world. The GLLP takes into account adult learning principles and is designed to be complementary to mid-career level individuals.

### CF and Development of Other Learning Programs

Ideally, when developing learning and training programs using the CF, the nine competencies will be taken in their entirety with activity areas in each competency reinforcing other competencies and providing a holistic approach to leadership development. Graduates of a laboratory leadership program designed around the CF should be required to complete and/or demonstrate proficiency in all competencies. However, each competency is designed to allow complementary learning opportunities for individual and specific competency development as needed. Competency-based program development allows separation of course materials into discrete units to address individual or group needs.

## Conclusion

The CF and upcoming implementation of the GLLP provide an innovative One Health approach to the development of laboratory leaders with the knowledge, skills and abilities to build bridges, enhance communication, foster collaboration and develop an understanding of existing synergies between the human, animal, environmental and other relevant health sectors. The CF represents the first global consensus on laboratory leadership competencies and provides structure for the development of leaders of the laboratory system by addressing gaps in current laboratory leadership learning programs. Fostering and mentoring laboratory leaders who understand the need for collaboration between all sectors of health laboratories will allow national health laboratory systems to grow and flourish through communication and coordination of activities. In a world where health events are becoming increasingly complex and can rarely be managed by a single sector alone, this CF was developed to enable leaders to set up multidisciplinary teams to respond to such health events as well as to promote a multidisciplinary vision for the management of laboratories and laboratory systems.

## Author Contributions

The authors are listed in alphabetical order on behalf of GLLP partners (APHL, CDC, ECDC, FAO, OIE, and WHO). All authors contributed to the conceptualization of this paper. AJ and PG led the competency framework conceptualization and development. AA and JI drafted the initial manuscript. EC, MH, BM, and BW contributed to the idea and manuscript framework, technical editing, and revisions.

### Conflict of Interest Statement

The authors declare that the research was conducted in the absence of any commercial or financial relationships that could be construed as a potential conflict of interest.
